# Reducing Central Line‐Associated Bloodstream Infections in the ICU: A Nurse‐Led Evidence Based Quality Improvement Initiative

**DOI:** 10.1002/nop2.70701

**Published:** 2026-07-15

**Authors:** Lu‐Xi Deng, Li‐Qing Yue, Li‐Na Zhang, Ping Zhong, Hong‐Man Wu, Jing‐Min Lai, Qing Yang, Lan Cao

**Affiliations:** ^1^ Teaching and Research Section of Clinical Nursing Xiangya Hospital, Central South University Changsha Hunan China; ^2^ Department of Critical Care Medicine Xiangya Hospital, Central South University Changsha Hunan China; ^3^ Department of Infection Control Center Xiangya Hospital, Central South University Changsha Hunan China

**Keywords:** central line‐associated bloodstream infection, central venous catheters, evidence‐based practice, quality improvement

## Abstract

**Aim:**

This quality improvement (QI) project aimed to develop, implement and evaluate a nurse‐led, evidence‐based central venous catheter (CVC) maintenance flowchart to reduce central line‐associated bloodstream infection (CLABSI) rates across five adult intensive care units (ICUs).

**Design:**

A prospective, pre‐post intervention QI project was conducted.

**Methods:**

A nurse‐led, multidisciplinary expert panel systematically reviewed the literature and graded evidence using the Joanna Briggs Institute (JBI) system. Recommendations were further evaluated for Feasibility, Appropriateness, Meaningfulness and Effectiveness (FAME), resulting in the development of an evidence‐based CVC Maintenance Flowchart. The flowchart was implemented across five ICUs comprising 116 beds and 267 nurses. Pre‐ and post‐intervention data on CLABSI rates (per 1000 CVC‐days), CVC utilisation and compliance with the maintenance bundle were collected and compared.

**Results:**

The mean CLABSI rate across five ICUs significantly decreased from 4.55 per 1000 CVC‐days in the pre‐intervention period to 1.68 per 1000 CVC‐days in the post‐intervention period (*p* < 0.001), representing a 63.2% reduction. Overall nurse adherence to the CVC maintenance flowchart components increased from a baseline average of 78% to 94.4% (*p* < 0.001). The central line utilisation ratio remained stable, indicating that the reduction in infections was attributable to improved care practices rather than decreased device usage.

**Conclusions:**

This nurse‐led QI initiative was associated with a substantial reduction in CLABSI rates among ICU patients and improved adherence to CVC maintenance practices. The project demonstrates that empowering frontline nurses with a structured implementation framework can bridge the gap between evidence and clinical practice, thereby enhancing patient safety in the ICU.

**Implications for Nursing Practice:**

Structured, nurse‐led implementation strategies help ICU nurses transform evidence‐based CVC maintenance recommendations into consistent bedside practices and may provide a practical framework for improving other nurse‐sensitive clinical outcomes.

**Patient or Public Contribution:**

Patients or members of the public were not involved in the design, conduct or reporting of this study.

## 1 Introduction and Background

Central venous catheters (CVCs) are indispensable devices in modern critical care. They provide reliable vascular access for medication administration, infusion of fluids and blood products and hemodynamic monitoring (Santoro et al. 2014; Wolf et al. 2013). However, the use of CVCs is associated with a high risk of complications, the most serious being central line‐associated bloodstream infections (CLABSIs), which rank among the most common and costly healthcare‐associated infections (HAIs) (Goudie et al. 2014). CLABSI can prolong hospital stays, increased morbidity and mortality, and place a substantial financial burden on the healthcare system (Oliveira et al. 2023; Wolf et al. 2013).

Fortunately, a large body of evidence shows that most CLABSIs are preventable (Spinks et al. 2025). Prevention strategies are commonly organised into ‘bundles’, which are sets of evidence‐based practices that can improve patient outcomes when implemented together (Wei et al. 2021). These bundles typically address both CVC insertion and subsequent daily maintenance. While proper insertion technique is essential to minimise initial contamination, maintenance practices are equally critical, as microorganisms can be introduced through the catheter hub or insertion site at any point during the device's dwell time (Wolf et al. 2013). Despite well‐established infection prevention bundles, ICUs continue to experience high CLABSI rates, pointing a persistent challenge in healthcare is the gap between the existence of evidence and its consistent implementation at the bedside (Chi et al. 2024; Ferrara and Albano 2018).

Nurses are central to closing this evidence‐practice gap. As the primary caregivers responsible for CVC maintenance, their knowledge, technical skills and adherence to protocols are essential for preventing CLABSI. Nurse‐led quality improvement (QI) initiatives that engage and support frontline staff may therefore contribute to better maintenance practices (Hussain et al. 2020; Turoldo et al. 2024). Translating complex guidelines into simple, actionable tools, such as checklists and flowcharts, may further help nurses apply complex guidelines more consistently at the bedside (Ling et al. 2016).

According to institutional infection surveillance data from the 3 years before the implementation of this QI project, the five specialised adult ICUs had a mean CLABSI rate of 5.90 per 1000 CVC‐days, indicating a persistently high infection burden. Preliminary observations suggested inconsistencies in CVC maintenance practices and a lack of a standardised, easily accessible protocol for bedside nurses. Therefore, this QI project was initiated with the primary aim of developing, implementing and evaluating a nurse‐led, evidence‐based CVC maintenance flowchart to reduce CLABSI rates and improve nursing practice across these high‐risk units.

## 2 Methods

### 2.1 Setting

This nurse‐led, evidence‐based QI project utilised a pre‐post, quasi‐experimental design. It was conducted across five adult ICUs (a 45‐bed Mixed ICU, a 20‐bed Neurosurgical ICU, a 15‐bed Neurological ICU, a 15‐bed Respiratory ICU and a 21‐bed Cardiac ICU) at Xiangya Hospital, Central South University, a large tertiary academic medical centre in Changsha, China. These units were selected due to their consistently high CLABSI rates. In total, the participating units comprised 116 beds and were staffed by 267 registered nurses. The project was structured in four distinct stages over a 12‐month period, including a 6‐month pre‐intervention baseline period and a 6‐month post‐intervention evaluation period.

This QI project was reviewed and approved by the Research Ethics Committee of Xiangya Hospital, Central South University (IRB No. 202203071) and registered at the Chinese Clinical Trial Registry (ChiCTR2200058673). Because the project was implemented as a clinical QI initiative using routinely collected, de‐identified infection surveillance, audit and nursing practice data, and involved no additional intervention beyond standard evidence‐based infection‐prevention practice, the requirement for individual informed consent was waived by the ethics committee. The results are reported in accordance with the Standards for QI Reporting Excellence (SQUIRE) version 2.0 guidelines (Ogrinc et al. 2016).

### 2.2 Intervention

#### 2.2.1 First Stage: Evidence Acquisition

A comprehensive literature review was conducted to identify current evidence‐based practices for CLABSI prevention in ICU settings. Databases including PubMed, CINAHL and the Cochrane Library were searched for clinical guidelines, systematic reviews, randomised controlled trials and primary studies published between 2005 and 2025. Search terms included ‘central line‐associated bloodstream infection’, ‘CLABSI’, ‘prevention’, ‘central venous catheter’, ‘CVC maintenance’ and ‘intensive care unit’.

The retrieved evidence was critically appraised and graded using the Joanna Briggs Institute (JBI) Levels of Evidence system, which classifies evidence from Level 1 (strongest, from systematic reviews of randomised controlled trials) to Level 5 (weakest, from expert opinion). Two panel members independently assessed each study and assigned an evidence level. Any disagreements were resolved through discussion or by a third panel member.

Following the evidence appraisal, the expert panel formulated a series of best practice recommendations. Each recommendation was then graded using the FAME (Feasibility, Appropriateness, Clinical Significance and Effectiveness) framework (Jordan et al. 2022). This process included a meeting with the expert panel and a broader group of 10 stakeholders, including frontline ICU nurses, a nursing supervisor, hospital administrators, an attending physician, a chief physician and a hospital infection control physician. During the meeting, each recommendation was discussed and stakeholders anonymously voted on the four FAME components. A recommendation was assigned a Grade A (Strong Recommendation) if there was broad consensus on its high clinical significance and effectiveness, with acceptable feasibility and appropriateness. A Grade B (Weak Recommendation) was assigned if there was less certainty or more variability in the evidence or stakeholder opinions regarding one or more FAME components. Ultimately, 24 items of evidence across seven dimensions were identified, as detailed in Table S1.

#### 2.2.2 Second Stage: Identifying Gaps and Barriers

A baseline assessment was performed to identify gaps between current practice and the best‐evidence recommendations. This involved:

1. *Nurse Knowledge Assessment:* A 30‐item questionnaire was developed based on the synthesised evidence. It was administered to all 267 nurses across the five ICUs to assess their baseline knowledge of CVC maintenance practices.
2. *Compliance Audits:* We applied the Mystery Shopping method (MSP), a structured observational approach historically used in retail and service industries to assess service processes and identify gaps in routine practice (Wilson 1998). This approach uses the perspective of patients or third‐party observers. It can help identify practical problems and procedural gaps that may not be found through routine supervision. In this project, both baseline and post‐intervention compliance audits were conducted by trained observers, including hospital infection control personnel and ICU nursing quality‐control members. All observers received standardised training on the checklist and participated in pilot observations before formal data collection to ensure consistent interpretation of each item. The checklist included key bundle elements such as hand hygiene, hub disinfection, dressing integrity and tubing changes.
3. *Barrier Analysis:* Stakeholder meetings involving nurses, department directors and physicians were held to discuss the main barriers affecting adherence to CVC maintenance practices. These barriers included the lack of standardised protocols, time constraints, workflow interruptions and inconsistent education.

#### 2.2.3 Third Stage: Implementation Strategies

**FIGURE 1:**
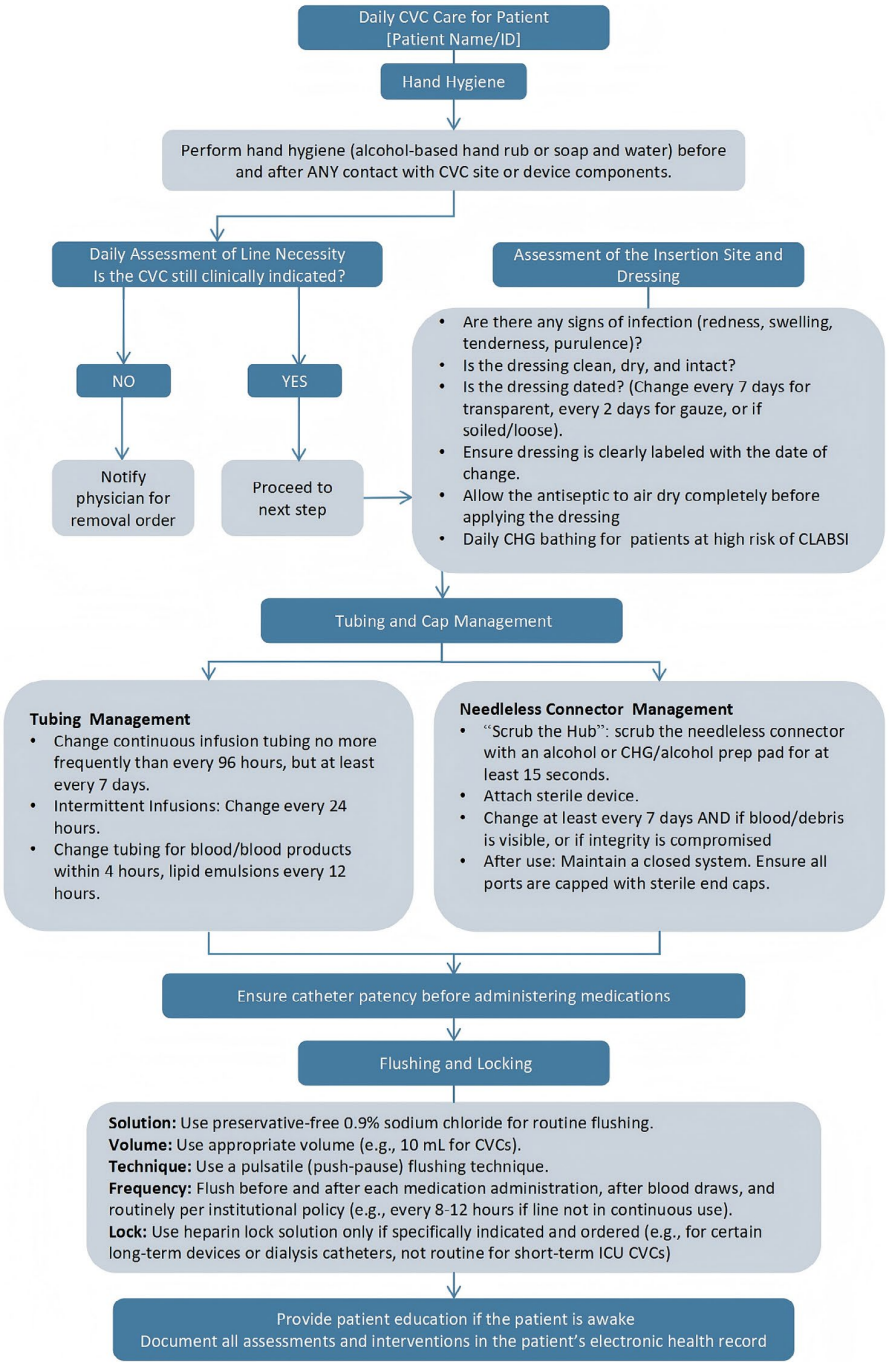
CVC maintenance flowchart for adult ICU patients. CHG, chlorhexidine gluconate; CLABSI, central line‐associated bloodstream infection; CVC, central venous catheter.

The intervention was multifaceted and was designed primarily to address the barriers identified during the baseline assessment:

1. *Education and Training:* Knowledge is a prerequisite for sustainable practice change. Educational interventions have also been shown to improve CLABSI‐prevention practices in adult ICUs (Foka et al. 2021). Therefore, a comprehensive training programme was developed for all ICU nursing staff. The programme included hands‐on training sessions covering the core components of CVC maintenance, hand hygiene and aseptic technique. CVC maintenance instructional videos were filmed and uploaded to a mobile‐based training platform, and all nurses were required to complete the relevant learning modules.
2. *CVC Maintenance Flowchart:* Based on the synthesised evidence from Stage 1, the expert panel developed a CVC Maintenance Flowchart (Figure 1). The core components of the flowchart included hand hygiene, daily assessment of line necessity, routine inspection of the insertion site and dressing, proper management of tubing and needleless connectors, and standardised protocols for flushing and locking the catheter (Buetti et al. 2020; Moureau and Flynn 2015; Perin et al. 2016; Yoshida et al. 2011). This tool synthesised the approved recommendations into a clear visual guide for daily nursing care. Laminated copies of the flowchart were placed at the bedside of each patient with a CVC.
3. *Audit and Feedback:* To ensure sustained adherence, an MSP method and Hospital Infection Control personnel conducted weekly audits on a random sample of patients with CVCs in each unit. Observers used a standardised checklist developed based on the evidence‐based CVC maintenance protocol to assess the implementation of key process indicators. The project team summarised the data at the department level and displayed results on public dashboard. Auditors provided real‐time feedback during each audit. Department leaders received detailed monthly performance reports, which provided a basis for identifying persistent barriers and collaboratively developing targeted action plans for further improvement.
4. *System‐Level Changes:* To support the new workflow, CVC dressing change kits were standardised and made readily available at the bedside. The morning handover system was modified to include mandatory daily assessment of CVC necessity, prompting clinicians to consider removal.

### 2.3 Data Collection and Analysis

The primary outcome was the CLABSI rate, expressed as the number of infections per 1000 CVC‐days. CLABSI cases were identified through prospective surveillance by the hospital's infection prevention department using the standardised case definitions from the Centers for Disease Control and Prevention's (CDC) National Healthcare Safety Network (NHSN) (Ling et al. 2016).

Secondary outcomes included: Central Line Utilisation Ratio: Calculated as the total number of central line‐days divided by the total number of patient‐days; Nursing Compliance Rate: The percentage of audited CVCs that were fully adherent to all applicable components of the maintenance flowchart. Data were collected from the weekly audits.

Data were analysed using SPSS Version 28.0. CLABSI rates were compared using a Poisson regression model to calculate an incidence rate ratio (IRR) and *p*‐value. As the nurse knowledge assessments were anonymous, knowledge‐test pass rates were compared at the aggregate level using the chi‐square test. Compliance rates were also compared using the chi‐square test. A *p* < 0.05 was considered statistically significant.

## 3 Results

A total of 3023 patients were included, with 1460 patients in the pre‐intervention period and 1563 patients in the post‐intervention period. In the 6‐month pre‐intervention period, the five ICUs recorded a total of 10,981 CVC‐days and 50 CLABSI events, for an aggregate baseline rate of 4.55 per 1000 CVC‐days. The baseline CVC utilisation ratio was 0.54. Direct observation of practice showed an average overall compliance with key maintenance elements of 78%, with particularly low adherence for Scrub the Hub (15 s) (63.3%) and daily assessment of line necessity (64.2%).

In the 6‐month post‐intervention period, the total number of CVC‐days was 10,138. A total of 17 CLABSI events were recorded, resulting in an aggregate rate of 1.68 per 1000 CVC‐days. This represents a statistically significant 63.2% reduction from the baseline rate (IRR = 0.37; 95% CI: 0.21–0.64; *p* < 0.001). The CVC utilisation ratio decreased slightly to 0.52. Detailed results are presented in Table 1.

**TABLE 1:** CLABSI rates and device utilisation on five ICUs during the pre‐ and post‐intervention phase.

Outcome measure	Pre‐intervention (6 months)	Post‐intervention (6 months)	Percent change	*p*
Total CVC‐days	10,981	10,138	−7.7%	N/A
Total CLABSI events	50	17	−62%	N/A
CLABSI rate (per 1000 CVC‐days)	4.55	1.68	−63.2%	< 0.001
CVC utilisation ratio	0.54	0.52	−3.7%	N/A

Abbreviations: CLABSI, central line‐associated bloodstream infection; CVC, central venous catheter.

Following the educational intervention, the knowledge‐test pass rate increased significantly from 60.7% to 93.6% (*p* < 0.001). Post‐intervention observations demonstrated a significant improvement in practice. The overall compliance with the CVC maintenance flowchart rose to an average of 94.4%. Adherence to specific components improved substantially, including hand hygiene (from 70.8% to 94.1%), scrub the Hub (from 63.3% to 93.1%) and daily assessment of line necessity (from 64.2% to 95%). A breakdown of compliance data is provided in Table 2.

**TABLE 2:** Nurse knowledge and compliance with CVC maintenance bundle pre‐ and post‐intervention.

Outcome measure	Pre‐intervention		Post‐intervention		*p*
	*n*	%	*n*	%	
Knowledge‐test pass rate (%)	162/267	60.7%	250/267	93.6%	< 0.001
Hand hygiene	119/168	70.8%	177/188	94.1%	< 0.001
Daily assessment of line necessity	70/109	64.2%	151/159	95.0%	< 0.001
Assess insertion site and dressing					
Dressing clean, dry, intact	75/109	68.8%	144/159	90.6%	< 0.001
Label the dressing with the date of change	86/109	78.9%	148/159	93.1%	< 0.001
Daily CHG bathing for high‐risk patients	34/51	66.7%	52/54	96.3%	< 0.001
Allow the antiseptic to air dry before dressing	52/80	65%	79/89	88.8%	< 0.001
Tubing and cap management					
Scrub the hub (15 s)	69/109	63.3%	148/159	93.1%	< 0.001
Change continuous infusion tubing (≤ 96 h, ≥ 7d)	78/98	79.6%	90/95	94.7%	< 0.001
Change needleless connector (≥ 7 days or as needed)	91/109	83.5%	145/159	91.2%	0.085
Change tubing for intermittent infusions (daily)	101/109	92.7%	158/159	99.4%	< 0.001
Change tubing for blood/blood products (≤ 4 h)	30/36	83.3%	40/42	95.2%	0.176
Change tubing for lipid emulsions (≤ 12 h)	31/40	77.5%	35/36	97.2%	< 0.001
Ensure catheter patency before administering medications	80/109	73.4%	145/159	91.2%	< 0.001
Flushing and locking					
Use preservative‐free 0.9% sodium chloride for flushing	53/62	85.5%	54/59	91.5%	0.451
Use 10 mL syringe for flushing	143/159	89.9%	106/109	97.2%	< 0.001
Each lumen flushed after every use	41/49	83.7%	54/56	96.4%	0.059
Use a pulsatile (push–pause) flushing technique	89/109	81.7%	156/159	98.1%	< 0.001
Apply normal saline/heparin solution for tube locking	101/109	92.7%	157/159	98.7%	< 0.001
Patient education and documentation					
Provide patient education if the patient is awake	14/22	68.2%	18/20	90%	0.101
Document all assessments and interventions	38/42	90.5%	54/56	96.4%	0.429
Mean compliance across maintenance bundle components (%)		78%		94.4%	

*Note:* Mean compliance across maintenance bundle components was calculated as the arithmetic mean of the percentage compliance values across all observed CVC maintenance‐bundle components listed in this table; the nurse knowledge‐test pass rate was excluded. Denominators differ across components because not all bundle components were applicable to every audited patient, catheter or clinical situation.

Abbreviations: CHG, chlorhexidine gluconate; CVC, central venous catheter.

## 4 Discussion

This QI project successfully demonstrated that a structured, nurse‐led initiative centred on an evidence‐based CVC maintenance flowchart can lead to a substantial and statistically significant reduction in CLABSI rates in a high‐risk, multi‐unit ICU setting. The 63.2% decrease in the CLABSI rate, from 4.55 to 1.68 per 1000 CVC‐days, is a clinically meaningful improvement that aligns with or exceeds the results of many similar initiatives reported in the literature (Chi et al. 2024; Spinks et al. 2025; Wei et al. 2021).

The success of this QI project can be attributed to several key factors. First, the CVC Maintenance Flowchart was developed through a careful evidence‐based process. The JBI and FAME frameworks were used to ensure that the final tool was effective, practical and suitable for our clinical setting. Second, the implementation strategy was multifaceted and focused on knowledge, skills and system support. The programme did not only provide a new protocol. It also offered targeted training, developed a clear visual flowchart and provided audit and feedback to support consistent adherence to the protocol. Moreover, the specific parts of the flowchart also addressed the main causes of CLABSI. Careful disinfection of the skin and infusion connectors aimed to reduce microbial migration through extraluminal and intraluminal routes, respectively (Wolf et al. 2013). The clear improvement in hub disinfection adherence, from 63.3% to 93.1%, may have contributed to the reduction in infections. Furthermore, Daily review of line necessity was also added to routine care. This process encouraged the clinical team to discuss each day whether the CVC was still needed. It could support timely catheter removal and reduce the total duration of catheter‐related risk (Jeffries et al. 2009; Sampson et al. 2023). Although our central line utilisation ratio remained stable, the documented daily review helped ensure that each line‐day was clinically justified and reinforced a culture of safety in catheter care.

A unique aspect of this project was its focus on CVC maintenance rather than insertion. This choice was based on our local clinical workflow. In many regions of China, CVC insertion is a specialised procedure performed by trained physicians under strict aseptic conditions, often with ultrasound guidance (Franco‐Sadud et al. 2019). Although critically important, CVC insertion is typically a single, time‐limited event. In contrast, CVC maintenance is a continuous process involving multiple nurses, repeated shift handovers and countless interactions with the catheter system. This prolonged exposure and high number of personnel interactions may increase the risk of contamination (Marschall et al. 2008). Therefore, we hypothesised that improving the repeated maintenance work performed by nurses across shifts could help reduce the risk of CLABSI.

### 4.1 Implications for Nursing Practice

This QI project shows that ICU nurses play an important role in preventing CLABSI through standardised daily CVC maintenance. The project findings suggest that evidence‐based recommendations are easier to implement when they are translated into clear and practical tools that fit the bedside nursing workflow. Improvements in adherence to hand hygiene, hub disinfection, dressing assessment, tubing and cap management, flushing and locking, and daily assessment of line necessity indicate that structured education, bedside reminders and regular audit‐feedback can enhance the consistency and reliability of CVC maintenance. By standardising these routine practices, nurse‐led interventions can help reduce practice variation across shifts and units and support more consistent catheter care.

### 4.2 Strengths and Limitations

This project has several strengths. It was conducted in multiple ICU settings within a single institution and involved a large number of nurses. It was also based on a rigorous and systematic methodology. In addition, the use of both process measures, such as adherence and a balancing measure, such as the central line utilisation ratio, provides a more comprehensive assessment of the intervention's impact. They also suggested that the improved outcomes were mainly related to better care practices, rather than simply less device use.

However, the study is not without limitations. The pre–post design without a concurrent control group means we cannot definitively exclude the influence of other temporal trends or hospital‐wide initiatives that may have contributed to the observed reduction in CLABSI rates. The project was conducted in a single academic medical centre, which may limit the generalisability of the findings to other settings, such as community hospitals. Finally, the Hawthorne effect‐whereby the act of observation influences behaviour‐may have contributed to the increased adherence rates. In addition, although observers received standardised training and completed pilot observations, formal inter‐rater reliability was not assessed; therefore, some observer‐related measurement variability may remain. We attempted to mitigate this by conducting audits over a long period and embedding them into routine practice through the unannounced audits.

## 5 Conclusion

This QI project successfully implemented a nurse‐led, evidence‐based CVC maintenance flowchart across five high‐risk ICUs, resulting in a substantial and statistically significant reduction in CLABSI rates. The initiative also markedly improved nurse adherence to best practices without negatively impacting central line utilisation. This study provides evidence that empowering frontline nurses with the right tools, knowledge and leadership structure is a powerful and effective strategy for translating evidence into practice, preventing healthcare‐associated infections and fundamentally improving patient safety in the critical care environment.

## Author Contributions

L.‐X.D. contributed to conceptualisation, methodology, project administration, data interpretation and drafting of the manuscript. L.‐Q.Y. contributed to quality control and supervision. L.‐N.Z. contributed to clinical interpretation and supervision. P.Z. and Q.Y. contributed to project implementation, data collection and quality control. H.‐M.W. and J.‐M.L. contributed to infection surveillance data collection and interpretation. L.C. contributed to study design, supervision and critical revision of the manuscript.

## Funding

The authors have nothing to report.

## Conflicts of Interest

The authors declare no conflicts of interest.

## Supporting information


**Table S1:** Summary of best evidence for central venous catheter maintenance in adult ICU patients.

## Data Availability

The data that support the findings of this quality improvement project are available from the corresponding author upon reasonable request. Due to institutional data protection requirements and the use of routinely collected clinical quality improvement and infection surveillance data, the dataset is not publicly available.
