# Supplementary material for: Reducing Central Line‐Associated Bloodstream Infections in the ICU: A Nurse‐Led Evidence Based Quality Improvement Initiative
Source: Nurs Open. 2026 Jul 15;13(7):e70701. doi: 10.1002/nop2.70701 (PMC13373459; doi:10.1002/nop2.70701)
Supplement: Supplementary file 1 — Table S1: Summary of best evidence for central venous catheter maintenance in adult ICU patients. [file NOP2-13-e70701-s001.docx]

Supplementary Table S1: Summary of Best Evidence for Central Venous Catheter Maintenance in Adult ICU Patients

| **Domain** | **Evidence Content** | **Level of Evidence** | **FAME Grade** |
| --- | --- | --- | --- |
| Assessment | 1. Assess the CVC insertion site daily for signs of infection (e.g., redness, pain, swelling, purulent discharge) through palpation over the intact dressing(Buetti et al., 2020). | 3 | A |
|  | 1. Assess the continued need for the CVC on a daily basis(Yoshida et al., 2011). | 3 | A |
| Hand Hygiene and Aseptic Technique | 3. Perform hand hygiene (using alcohol-based hand rub or soap and water) immediately before and after any contact with the CVC, its dressing, or the infusion system(Perin et al., 2016). | 1 | A |
|  | 4.Maintain a sterile field and use sterile technique, including sterile gloves and mask, for all dressing changes(Gorski, 2024). | 4 | A |
| Catheter Site and Dressing Care | 5.Use a >0.5% chlorhexidine gluconate (CHG) with alcohol preparation for skin antisepsis during dressing changes(Deng et al., 2025). | 1 | A |
|  | 6.Allow the antiseptic to air dry completely according to the manufacturer’s instructions before applying the dressing(Gorski, 2024). | 1 | A |
|  | 7.Consider daily skin cleansing with CHG-impregnated cloths for all ICU patients to reduce the overall bioburden and risk of CLABSI(Afonso, Blot, & Blot, 2016). | 1 | A |
|  | 8.Use a sterile, transparent, semipermeable polyurethane dressing to cover the puncture site with the puncture point as the center; Tension-free fixation with sterile transparent dressing; Date of change should be marked on the outside of the dressing(Dang, Li, & Tian, 2019). | 1 | A |
|  | 9.Change sterile gauze every 2 days and transparent dressings every 7 days, or immediately if the dressing is damp, loosened, or visibly soiled(Timsit et al., 2009). | 1 | A |
|  | 10.Use a chlorhexidine-impregnated dressing for ICU patients with CVCs, particularly in units with high CLABSI rates(Timsit et al., 2009). | 1 | B |
| System and Tubing Management | 11.Decontaminate catheter hubs, needleless connectors, and injection ports by scrubbing with a 2% chlorhexidine in 70% alcohol pred pad for at least 15 seconds and allowing it to dry completely before each access(Moureau & Flynn, 2015). | 1 | A |
|  | 12. Change administration sets used for continuous infusions no more frequently than every 96 hours, but at least every 7 days(Ullman et al., 2013). | 1 | A |
|  | 13.Change administration sets after 24 hours of intermittent infusion or upon completion of daily infusion. Change tubing for blood/blood products every 4 hours. Change tubing for lipid emulsions every 12 hours(Chinese Society Of Critical Care, 2025). | 5 | A |
|  | 14. Minimize CVC access and keep the infusion system closed as much as possible(Templeton et al., 2008). | 5 | A |
| Catheter Patency and Flushing | 15.Flush each CVC lumen with preservative-free 0.9% sodium chloride before and after each medication administration to confirm patency and prevent admixture of incompatible drugs(Zhou et al., 2025). | 2 | A |
|  | 16.Use a pulsatile (push-pause) flushing technique with a 10 mL or larger syringe to create turbulence within the lumen, which helps to remove deposits from the catheter wall(Wang et al., 2024). | 5 | B |
|  | 17.Lock intermittently used lumens with preservative-free 0.9% sodium chloride. Avoid the routine use of heparin-containing solutions(Sotnikova et al., 2020). | 2 | A |
| Education and Competency | 18.Ensure all healthcare personnel involved in CVC maintenance receive comprehensive education on CLABSI prevention and are competent in all related procedures(Foka et al., 2021). | 1 | A |
|  | 19.Periodically assess the competency of staff in performing CVC maintenance procedures through direct observation or simulation(Wang et al., 2024). | 4 | A |
|  | 20.Provide regular performance feedback to ICU staff on adherence rates to CVC maintenance bundles and CLABSI rates(Longmate et al., 2011). | 3 | B |
| Organizational and System Support | 21.Implement CVC maintenance practices as a “bundle” of care, rather than as individual, isolated interventions(Perin et al., 2016). | 1 | A |
|  | 22.Empower all members of the healthcare team, including nurses, to stop a procedure if breaches in aseptic technique are observed(Franco, Delgadillo, & Siller, 2024). | 5 | A |
|  | 23.Ensure ready availability of all necessary supplies for CVC maintenance, such as CVC dressing kits and appropriate antiseptics(Cervera-Jackson et al., 2025). | 5 | B |
|  | 24.Foster strong institutional and leadership commitment to CLABSI prevention through resource allocation, policy development, and visible support for quality improvement initiatives(Jeffries et al., 2009). | 5 | B |

*Note*: CHG, chlorhexidine gluconate; CLABSI, central line-associated bloodstream infection; CVC, central venous catheter; ICU, intensive care unit; JBI, Joanna Briggs Institute; FAME, Feasibility, Appropriateness, Meaningfulness, and Effectiveness.

**References:**

Afonso, E., Blot, K., & Blot, S. (2016). Prevention of hospital-acquired bloodstream infections through chlorhexidine gluconate-impregnated washcloth bathing in intensive care units: a systematic review and meta-analysis of randomised crossover trials. *Euro Surveill, 21*(46). doi:10.2807/1560-7917.Es.2016.21.46.30400

Buetti, N., Ruckly, S., Lucet, J. C., Bouadma, L., Garrouste-Orgeas, M., Schwebel, C., Timsit, J. F. (2020). Local signs at insertion site and catheter-related bloodstream infections: an observational post hoc analysis using individual data of four RCTs. *Crit Care, 24*(1), 694. doi:10.1186/s13054-020-03425-0

Cervera-Jackson, R., McCready, P., Ireland, S., Riphagen, S., Adalat, S., Adams, A., & Plater, T. (2025). Human factors and systems approach to environmental sustainability in critical care units across a large multi-site English health care organization. *Nurs Crit Care, 30*(1), 71-74. doi:10.1111/nicc.13252

Chinese Society Of Critical Care, M. (2025). [Practice guideline on the prevention and treatment of central line associated bloodstream infection in 2025]. *Zhonghua Wei Zhong Bing Ji Jiu Yi Xue, 37*(3), 193-220. doi:10.3760/cma.j.cn121430-20250301-00199

Dang, F. P., Li, H. J., & Tian, J. H. (2019). Comparative efficacy of 13 antimicrobial dressings and different securement devices in reducing catheter-related bloodstream infections: A Bayesian network meta-analysis. *Medicine (Baltimore), 98*(14), e14940. doi:10.1097/md.0000000000014940

Deng, A., Xiong, F., & Ren, Q. (2025). Chlorhexidine solutions are more effective than povidone-iodine solutions as skin disinfectants for the prevention of intravascular catheter-related infections: A meta-analysis. *Sci Rep, 15*(1), 10657. doi:10.1038/s41598-025-92476-w

Foka, M., Nicolaou, E., Kyprianou, T., Palazis, L., Kyranou, M., Papathanassoglou, E., & Lambrinou, E. (2021). Prevention of Central Line-Associated Bloodstream Infections Through Educational Interventions in Adult Intensive Care Units: A Systematic Review. *Cureus*. doi:10.7759/cureus.17293

Franco, C., Delgadillo, C., & Siller, K. J. N. C. O. A. J. (2024). Factors associated with central venous catheter infection in critically ill patients. *10*(3), 104-108.

Gorski, L. A. (2024). Update: The 2024 Infusion Therapy Standards of Practice. *Home Healthc Now, 42*(4), 198-205. doi:10.1097/nhh.0000000000001270

Jeffries, H. E., Mason, W., Brewer, M., Oakes, K. L., Muñoz, E. I., Gornick, W., Jarvis, W. R. (2009). Prevention of central venous catheter-associated bloodstream infections in pediatric intensive care units: a performance improvement collaborative. *Infect Control Hosp Epidemiol, 30*(7), 645-651. doi:10.1086/598341

Longmate, A. G., Ellis, K. S., Boyle, L., Maher, S., Cairns, C. J., Lloyd, S. M., & Lang, C. (2011). Elimination of central-venous-catheter-related bloodstream infections from the intensive care unit. *BMJ Qual Saf, 20*(2), 174-180. doi:10.1136/bmjqs.2009.037200

Moureau, N. L., & Flynn, J. (2015). Disinfection of Needleless Connector Hubs: Clinical Evidence Systematic Review. *Nurs Res Pract, 2015*, 796762. doi:10.1155/2015/796762

Perin, D., Erdmann, A., Higashi, G., & Sasso, G. (2016). Evidence-based measures to prevent central line-associated bloodstream infections: a systematic review. *Rev Lat Am Enfermagem, 24*(0). doi:10.1590/1518-8345.1233.2787

Sotnikova, C., Fasoi, G., Efstathiou, F., Kaba, E., Bourazani, M., & Kelesi, M. (2020). The Efficacy of Normal Saline (N/S 0.9%) Versus Heparin Solution in Maintaining Patency of Peripheral Venous Catheter and Avoiding Complications: a Systematic Review. *Mater Sociomed, 32*(1), 29-34. doi:10.5455/msm.2020.32.29-34

Templeton, A., Schlegel, M., Fleisch, F., Rettenmund, G., Schöbi, B., Henz, S., & Eich, G. (2008). Multilumen central venous catheters increase risk for catheter-related bloodstream infection: prospective surveillance study. *Infection, 36*(4), 322-327. doi:10.1007/s15010-008-7314-x

Timsit, J. F., Schwebel, C., Bouadma, L., Geffroy, A., Garrouste-Orgeas, M., Pease, S., Lucet, J. C. (2009). Chlorhexidine-impregnated sponges and less frequent dressing changes for prevention of catheter-related infections in critically ill adults: a randomized controlled trial. *Jama, 301*(12), 1231-1241. doi:10.1001/jama.2009.376

Ullman, A. J., Cooke, M. L., Gillies, D., Marsh, N. M., Daud, A., McGrail, M. R., Rickard, C. M. (2013). Optimal timing for intravascular administration set replacement. *Cochrane Database Syst Rev, 2013*(9), Cd003588. doi:10.1002/14651858.CD003588.pub3

Wang, W., Fu, Q., Shen, W., Xu, Y., Wang, L., Ma, X., Gu, Y. (2024). Using the i-PARIHS theoretical framework to develop evidence implementation strategies for central venous catheter maintenance: a multi-site quality improvement project. *JBI Evidence Implementation, 22*(2), 195-204. doi:10.1097/xeb.0000000000000418

Yoshida, J., Ishimaru, T., Kikuchi, T., Matsubara, N., & Asano, I. (2011). Association between risk of bloodstream infection and duration of use of totally implantable access ports and central lines: a 24-month study. *Am J Infect Control, 39*(7), e39-43. doi:10.1016/j.ajic.2010.11.013

Zhou, T., Li, C., Wang, Z., Yang, M., He, X., & Hu, Y. (2025). Evidence‐Based Practice in Maintenance of Central Venous Catheters Among Intensive Care Unit Nurses: A Cross‐Sectional Multi‐Center Study. *J Clin Nurs*. doi:10.1111/jocn.17692
